# Gratitude buffers the effects of stressful life events and deviant peer affiliation on adolescents’ non-suicidal self-injury

**DOI:** 10.3389/fpsyg.2022.939974

**Published:** 2022-09-30

**Authors:** Chang Wei, Yu Wang, Tao Ma, Qiang Zou, Qian Xu, Huixing Lu, Zhiyong Li, Chengfu Yu

**Affiliations:** ^1^Research Center for Rural Educational and Cultural Development of Key Research Base of Humanities and Social Sciences in Hubei Province, School of Education, Hubei University of Science and Technology, Xianning, China; ^2^School of Psychology, South China Normal University, Guangzhou, China; ^3^Liuxiang Junior High School, Zhuhai, China; ^4^College of Education Science, Hubei Normal University, Huangshi, China; ^5^School of Education, Guangzhou University, Guangzhou, China

**Keywords:** stressful life events, gratitude, deviant peer affiliation, non-suicidal self-injury, adolescents

## Abstract

Although stressful life events have been shown to be a key risk factor for adolescent NSSI, the potential mediators and moderators of this relationship are unclear. Based on the social development theory and the organism-environment interaction model, we tested whether the link between stressful life events and adolescent NSSI was explained in part by deviant peer affiliation, and whether this process was buffered by gratitude. Chinese adolescents (*N* = 854; *M_*age*_* = 16.35; 68.50% female) anonymously completed questionnaires to assess the study variables. The present study demonstrated that stressful life events was linked to NSSI in part because of deviant peer affiliation, and high gratitude was a key protective factor to buffer this indirect effect. Teaching gratitude may be a helpful component of prevention and intervention programs to reduce adolescent NSSI.

## Introduction

Non-suicidal self-injury (NSSI) is becoming a more prevalent and serious health problem among adolescents (Swannell et al., 2014; Cipriano et al., 2017; Hepp et al., 2020; Wu et al., 2021). NSSI refers to the deliberate and direct destruction of one’s own body tissue without suicidal intention, that is likely to induce bleeding, bruising, or pain, most commonly by cutting, burning, stabbing, hitting, or excessive rubbing (Nock, 2010; American Psychiatric Association, 2022). In 2014, the estimated prevalence of adolescent NSSI worldwide was 17.2% (Swannell et al., 2014). In the most recent study, Wu et al. (2021) found in a sample of 1,724 Chinese adolescents that 386 (22.7%) reported at least one act of NSSI in the past 6 months. Moreover, the development of NSSI during adolescence is an important predictor of future suicidal behaviors (Guan et al., 2012; Mars et al., 2019).

Because of the prevalence and seriousness of NSSI among today’s adolescents, it is important to identify risk and protective factors that can be taken into account in prevention and intervention. Using the social development model (Hawkins and Weis, 1985) and the organism-environment interaction model (Cummings et al., 2002) as a conceptual framework, and building on the empirical research to date, we developed and tested a model in which stressful life events are a risk factor for adolescent NSSI, and this risk is explained in part by deviant peer affiliation. We also tested whether the adolescent’s gratitude could buffer the effects of this risk process.

### Stressful life events and adolescent NSSI

Stressful life events (such as family conflicts, conflict with teachers, classmate disputes, economic distress, death of relatives, and failure in examination) are important predictors of adolescent NSSI (Gao et al., 2020). Nock’s (2010) integrated theoretical model of NSSI proposes that NSSI is an effective means of regulating social situations, which has a function of interpersonal negative reinforcement (e.g., NSSI facilitates stop from family conflicts).

Consistent with this theory (Nock, 2010), adolescents who have experienced stressful life events may engage in NSSI as a stress regulation strategy. A growing body of evidence has shown that stressful life events increased the risk of NSSI among adolescents (Tang et al., 2016; Cipriano et al., 2020; Gao et al., 2020; Steinhoff et al., 2020). Steinhoff et al. (2020) found in a longitudinal study of 1,482 adolescents that stressful life events were consistently positively associated with NSSI in peer context. Similarly, in a longitudinal study of 279 Chinese adolescents, Gao et al. (2020) found that stressful life events were associated with NSSI 6 and 12 months later. Steinhoff et al. (2020) and Gao et al. (2020) provide strong evidence of a direct link between stressful life events and adolescent NSSI.

### Deviant peer affiliation as a potential mediator

Deviant peer affiliation is defined as selectively affiliating with peers who show behaviors such as drinking, smoking, cannabis use, or truancy (Fergusson and Horwood, 1999; Fergusson et al., 2003). Deviant peer affiliation is an important proximal process affecting adolescents’ problem behaviors in China (Zang et al., 2022). Gao et al. (2019) assert that deviant peer affiliation can directly or indirectly affect adolescents’ problem behaviors through peer group pressure, role modeling, and behavioral reinforcement, reflecting a process of socialization.

Previous studies have confirmed that deviant peer affiliation is an important risk factor for Chinese adolescents’ aggressive behavior (Lin et al., 2020), tobacco and alcohol use (Sun et al., 2021), and internet gaming addiction (Tian et al., 2019). Moreover, several studies have shown that behaviors such as drinking, internet addiction, and aggressive behavior were positively correlated with NSSI among Chinese adolescents (Zhang et al., 2019; Pang and Wang, 2020; Gui et al., 2021). For example, Gui et al. (2021) found that drinking was positively associated with NSSI in a simple of 9,247 Chinese adolescents. Therefore, we assume that deviant peer affiliation should increase this risk of NSSI. Indeed, a previous study showed that deviant peer affiliation was positively correlated with NSSI among Chinese adolescents (Wei et al., 2021). In addition, the social development model (Hawkins and Weis, 1985) postulates that families, schools, peers, and communities all contribute to individual development. According to the social development model (Hawkins and Weis, 1985), adolescents who interact with deviant peers are more likely to develop risk behaviors (such as NSSI).

Several studies have documented that adolescents who experience stressful life events are more likely to show deviant peer affiliation (Zhu et al., 2016; Chen et al., 2020; Lin et al., 2020). This may be because stressful life events increase adolescents’ social alienation, which in turn contribute to subsequent interactions with deviant peers (Rudolph et al., 2013). Previous research also demonstrated that deviant peer affiliation could mediate the association between stressful life events and risk behaviors (Lansford et al., 2014; Dou et al., 2020; Lin et al., 2020). For example, in a longitudinal study, Dou et al. (2020) found that deviant peer affiliation mediated the relationship between negative parenting and risk-taking behavior in adolescents.

### Gratitude as a moderator

Gratitude is a traditional virtue in China. This concept is strongly communicated from one generation to the next. Chinese adolescents have been socialized to feel and express gratitude since childhood (Yu et al., 2010). Previous studies have confirmed that gratitude is an important protective factor for Chinese adolescents’ risk behavior (Yu et al., 2010; Zhou et al., 2014; Sun et al., 2019). Gratitude refers to a generalized tendency to recognize and respond with grateful emotion to the roles of others’ benevolence in their own positive experiences and outcomes (McCullough et al., 2002). The broaden-and-build theory (Fredrickson, 1998, 2001, 2004) proposes that gratitude helps individuals build their enduring personal resources. These include physical resources, social, and psychological resources. According to this theory, gratitude improves adolescents’ adaptability in the face of adversity. Based on this theory, we assume that more gratitude should reduce this risk of deviant peer affiliation and NSSI. Indeed, previous studies have shown that gratitude was negatively correlated with deviant peer affiliation (Zhou et al., 2014; Lin et al., 2015) and NSSI (Jiang et al., 2020) among Chinese adolescents.

In the current study we were interested not in this direct link between gratitude and NSSI, but rather in role of gratitude as a buffer against the effects of environmental risk factors. The organism-environment interaction model (Cummings et al., 2002) provides a useful framework for conceptualizing gratitude as a moderator. According to this model, individual characteristics interact with environment factors to shape behavior. In the current study we were interested in the interaction between gratitude as an individual difference, and stressful life events as environmental risk factors, in predicting NSSI.

In the proposed moderated mediation model, we test gratitude as a moderator of both links in the mediated pathway by which stressful life events are associated with NSSI by way of deviate peer affiliation. First, gratitude may weaken the relationship between stressful life events and deviant peer affiliation. Based on the broaden-and-build theory (Fredrickson, 1998, 2001, 2004), gratitude as a protective factor may weaken the relationship between stressful life events and deviant peer affiliation. Indeed, gratitude has been shown to buffer the risk effect of stressful life events on adolescents’ outcomes (Zhou et al., 2014; Lo et al., 2017; Duprey et al., 2020). For example, in a sample of 1,194 Chinese adolescents, Zhou et al. (2014) found that the high level of gratitude buffered the negative effect of peer victimization on problematic online game use.

Second, gratitude may weaken the risk effect of deviant peer affiliation on adolescent NSSI. Although this possibility is supported by the broaden-and-build theory (Fredrickson, 1998, 2001, 2004), there are few empirical studies that are relevant to this question. Prior study have shown that adolescents who affiliate with deviant peers are more likely to engage in NSSI (Wei et al., 2021), and there is evidence that gratitude is associated with lower NSSI (Jiang et al., 2020). However, it is unclear how these two variables (deviant peer affiliation and gratitude) might interact to predict NSSI. Very indirect evidence comes from one study by Lo et al. (2017). In a sample of 447 Chinese children, Lo et al. (2017) found that the high level of gratitude buffered the negative effect of different parenting style (such as low level of warmth, low level of autonomy granting, and high level of dominating) on children’s suicidal ideation.

### The present study

**FIGURE 1:**
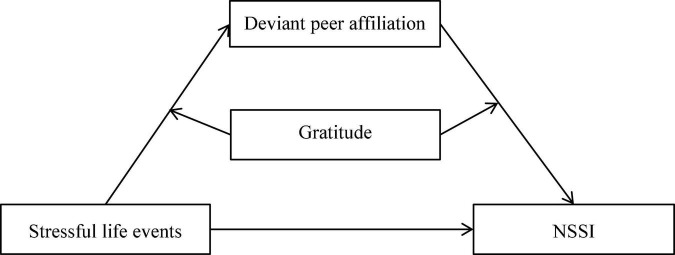
The proposed mediated moderation model. NSSI, non-suicidal self-injury.

Grounded in the social development model of delinquency prevention (Hawkins and Weis, 1985) and the organism-environment interaction model (Cummings et al., 2002), this study tested a moderated mediation model to elucidate predictors of adolescent NSSI. this study investigated whether deviant peer affiliation mediates the relation between stressful life events and adolescent NSSI and whether this indirect link is moderated by gratitude. The proposed model can be seen in Figure 1.

We proposed the following hypotheses: (1) deviant peer affiliation will mediate the association between stressful life events and adolescent NSSI; (2) gratitude will weaken the indirect relationship between stressful life events and adolescent NSSI through deviant peer affiliation. Specifically, gratitude will buffer the effect of stressful life events on deviant peer affiliation, and it will also buffer the effect of deviant peer affiliation on NSSI.

## Materials and methods

### Participants

We recruited participants from a secondary vocational and technical school in the provinces of Hubei in China. The sample included 854 adolescents (*M_age_* = 16.35; *SD* = 1.15); among them, 269 were boys (31.50%), 585 were girls (68.50%). Of the 854 adolescents in the general sample of students, 26.8% endorsed one or more NSSI behaviors in the past year. Among this sample, 67.40% of participants’ fathers and 74.70% of their mothers had not completed secondary vocational and technical school or a high school.

This research was approved by the Ethics in Human Research Committee at the university with which the author is affiliated. The questionnaires were administered by college teachers and trained college students. All the data collected are anonymous.

### Measures

#### Stressful life events

Stressful life events were measured with the Adolescent Life Events Scale (Liu et al., 1997), which includes 27 items (such as “family conflicts”, “tense relationship with teachers”, “classmate disputes”, “economic distress”, “death of relatives”, and “failure in examination”). Previous studies found that the questionnaire had good reliability in a sample of Chinese adolescents (Huang et al., 2017). The scale contained interpersonal relationships, learning stress, punishment, loss, health adjustment, and other stress. Adolescents were asked to report whether or not they had experienced each event in the past year. Each item was scored with 6 points (0 = none *to* 5 = very serious). The higher the score showed the higher and serious stressful life events. Cronbach’s alpha in this study was 0.94.

#### Gratitude

We assessed gratitude with the Chinese version (Wei et al., 2011) of the Gratitude Questionnaire (McCullough et al., 2002). Previous studies found that the questionnaire had good reliability in testing Chinese adolescents (Lin et al., 2021). Adolescents were asked to report their feeling grateful. This questionnaire included six items (such as “There are a lot of people in my life that I am grateful for”), each rated from (1 = absolutely disagree *to* 7 = absolutely agree). A higher average score reflected a higher level of gratitude. Cronbach’s alpha in this study was 0.79.

#### Deviant peer affiliation

We assessed deviant peer affiliation with the Deviant Peer Affiliation Scale (Zhou et al., 2014). Previous studies found that the scale had good reliability in testing Chinese adolescents (Su et al., 2017). Adolescents were asked to report whether they had friends with the deviant behaviors, and how often did you get along with them in the last week. The scale contained 16 items (such as “fight”, “play truant or truant”), each item was scored with 5 points (1 = none *to* 5 = six or more). A higher average score indicated the more affiliation with deviant peers. Cronbach’s alpha in this study was 0.87.

#### Non-suicidal self-injury

NSSI is measured with the Non-Suicidal Self-Injury (NSSI) Scale (You et al., 2013; You and Lin, 2015), which includes seven items such as self-cutting, burning, biting and so on. Previous studies (Yu et al., 2020) have shown that the reliability of the scale is good. Each item was scored with 4 points (1 = never *to* 4 = six or more times). Cronbach’s alpha in this study was 0.84.

#### Control variables

Much of the research on NSSI has focused on adolescents, who show a higher rate of NSSI than other age groups (for a review, see Swannell et al., 2014). Evidence generally indicates that females are at higher risk for NSSI among adolescents (for a review, see Rahman et al., 2021). In addition, a recent study showed that family income per month was positively related to NSSI among Chinese adolescents (Wang and Liu, 2022). Therefore, we controlled gender, age, and family per capita monthly income.

### Statistical analyses

SPSS 21.0 was used for the descriptive statistical analyses. Mediation and moderation effects were tested with Mplus 8.3. Missing data were handled via the full information maximum-likelihood (FIML) estimation method. This method does not use substitution to fill in the missing data, but uses known information to estimate parameters iteratively. We used bootstrapping with 5,000 iterations to verify the significance of the paths. Model fit is considered good when χ^2^/*df* < 5, CFI > 0.90, TLI > 0.90, RMSEA < 0.08, and SRMR < 0.08 (Hoyle, 2012)

## Results

### Preliminary analyses

The measure of NSSI included the following categories: self-cutting (*n* = 133), burning (*n* = 24), biting (*n* = 105), punching (*n* = 34), scratching skin (*n* = 91), inserting objects into the nail or skin (*n* = 38), and banging the head or other parts of the body against the wall (*n* = 57). The means, standard deviations, and correlation coefficients for all variables are displayed in Table 1. Stressful life events (*r* = 0.21, *p* < 0.001) and deviant peer affiliation (*r* = 0.35, *p* < 0.001) were both positively correlated with NSSI. The stressful life events variable was also positively correlated with deviant peer affiliation (*r* = 0.21, *p* < 0.001). Moreover, gratitude was negatively correlated with both deviant peer affiliation (*r* = –0.07, *p* < 0.05) and NSSI (*r* = –0.19, *p* < 0.001).

**TABLE 1:** Descriptive statistics and correlations for all variables.

Variable	1	2	3	4	5	6	7
1.Gender							
2.Age	0.07*						
3.Income	−0.02	–0.04					
4.SLE	−0.05	−0.09*	−0.04				
5.Gratitude	−0.04	0.07*	0.05	−0.09*			
6.DPA	0.19***	0.00	−0.02	0.21***	–0.07*		
7.NSSI	−0.04	−0.05	0.02	0.21***	−0.19***	0.35***	
*Mean*	0.31	16.35	2.54	1.33	5.17	1.33	1.10
*SD*	0.47	1.15	1.68	0.79	1.05	0.45	0.27

*p < 0.05. ***p < 0.001. Gender was dummy coded as 1 = male, 0 = female. SLE = stressful life events; DPA = deviant peer affiliation; NSSI = non-suicidal self-injurious behavior.

### Mediation effect of deviant peer affiliation

**FIGURE 2:**
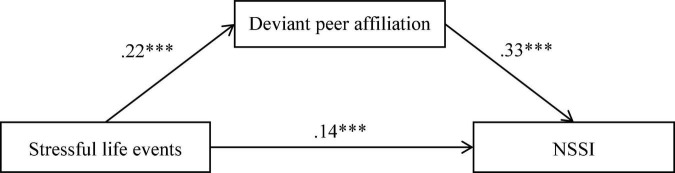
Model of the mediating role of deviant peer affiliation between stressful life events and NSSI. NSSI, non-suicidal self-injury. For simplicity, the effects of demographics on deviant peer affiliation and NSSI are not displayed. ^∗∗∗^*p* < 0.001.

The hypothesized mediation model showed a good fit to the data, χ^2^/*df* = 2.92, CFI = 0.96, TLI = 0.91, RMSEA = 0.05, and SRMR = 0.03. Figure 2 displays the results of model testing. Stressful life events positively predicted deviant peer affiliation (*b* = 0.22, *SE* = 0.03, *p* < 0.001) and positively predicted NSSI (*b* = 0.14, *SE* = 0.03, *p* < 0.001). Deviant peer affiliation positively predicted NSSI (*b* = 0.33, *SE* = 0.07, *p* < 0.001). Of the paths involving control variables, gender (dummy coded as 1 = male, 0 = female) was significantly related to deviant peer affiliation (*b* = 0.21, *SE* = 0.04, *p* < 0.001) and NSSI (*b* = –0.09, *SE* = 0.04, *p* < 0.05). Bootstrapping analyses showed that deviant peer affiliation partially mediated the pathway from stressful life events to NSSI (indirect effect = 0.07, *SE* = 0.02, 95% CI = [0.04, 0.12]).

### Moderated mediation

**FIGURE 3:**
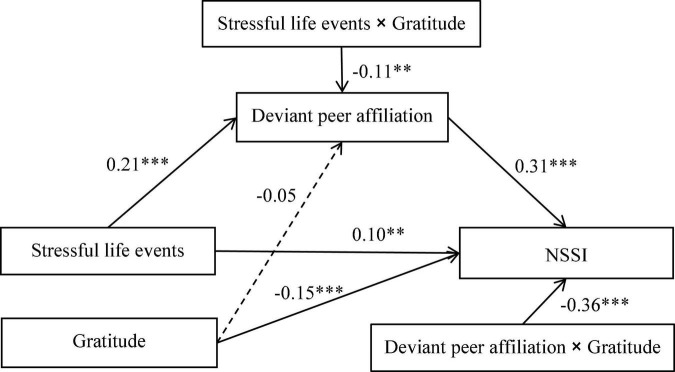
Model of the moderating role of gratitude on the indirect relationship between stressful life events and NSSI. NSSI, non-suicidal self-injury. For simplicity, the effects of demographics on deviant peer affiliation and NSSI are not displayed. ^∗∗^*p* < 0.01. ^∗∗∗^*p* < 0.001.

The moderated mediation model represented in Figure 3 had a good fit to the data: χ^2^/*df* = 2.34, CFI = 0.96, TLI = 0.94, RMSEA = 0.04, and SRMR = 0.04. Stressful life events (*b* = 0.10, *SE* = 0.04, *p* < 0.01), deviant peer affiliation (*b* = 0.31, *SE* = 0.06, *p* < 0.001) and gratitude (*b* = –0.15, *SE* = 0.03, *p* < 0.001) were significantly associated with NSSI. The stressful life events variable was also significantly associated with deviant peer affiliation (*b* = 0.21, *SE* = 0.03, *p* < 0.001), but the association between gratitude and deviant peer affiliation was not significant (*b* = –0.05, *SE* = 0.04, *p* > 0.05). More importantly, gratitude significantly moderated the effect of stressful life events on deviant peer affiliation (*b* = –0.11, *SE* = 0.04, *p* < 0.01), and the effect of deviant peer affiliation on NSSI (*b* = –0.36, *SE* = 0.08, *p* < 0.001). Of the paths involving control variables, gender (dummy coded as 1 = male, 0 = female) was significantly related to deviant peer affiliation (*b* = 0.22, *SE* = 0.04, *p* < 0.001) and NSSI (*b* = –0.07, *SE* = 0.04, *p* < 0.05).

**FIGURE 4:**
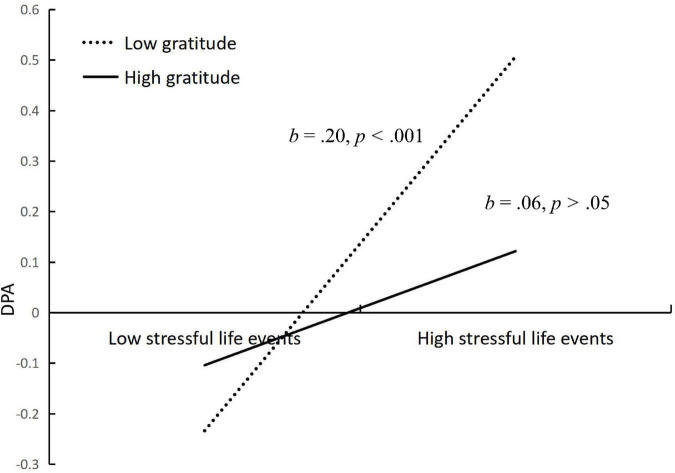
Interactive effect of stressful life events and gratitude on deviant peer affiliation.DPA, deviant peer affiliation. Gratitude is graphed for two groups of participants: high gratitude (1 SD above the mean) and low gratitude (1 SD below the mean).

**FIGURE 5:**
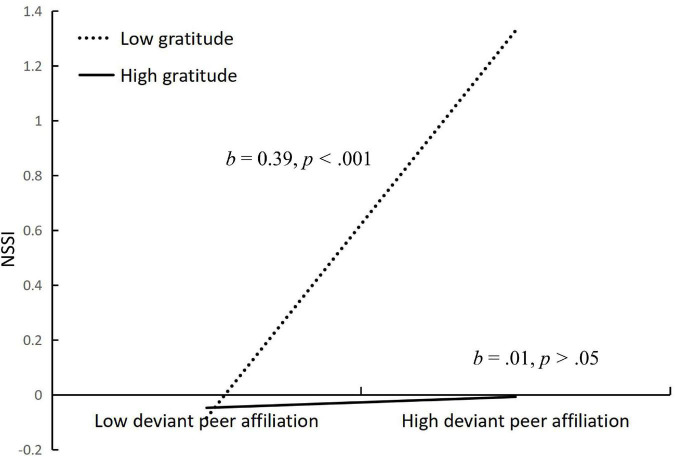
Interactive effect of deviant peer affiliation and gratitude on NSSI. NSSI, non-suicidal self-injury. Gratitude is graphed for two groups of participants: high gratitude (1 SD above the mean) and low gratitude (1 SD below the mean).

We conducted simple slopes tests to interpret the moderating effects of gratitude in the proposed model. As depicted in Figure 4, shows the relationship between stressful life events and deviant peer affiliation at low (at 1 SD below the mean) and high (at 2 SD above the mean) levels of gratitude. To be specific, the relation between stressful life events and deviant peer affiliation was significant for adolescents with lower gratitude (*b* = 0.20, *SE* = 0.03, *p* < 0.001), but was non-significant for those with higher gratitude (*b* = 0.06, *SE* = 0.03, *p* > 0.05). Similarly, as depicted in Figure 5, shows the relationship between deviant peer affiliation and NSSI at low (at 1 SD below the mean) and high (at 2 SD above the mean) levels of gratitude. To be specific, the relation between deviant peer affiliation and NSSI was significant for adolescents with lower gratitude (*b* = 0.39, *SE* = 0.02, *p* < 0.001), but was non-significant for those with higher gratitude (*b* = 0.01, *SE* = 0.03, *p* > 0.05). In sum, gratitude reduced the strength of the association between stressful life events and deviant peer affiliation, and also reduced the strength of the association between deviant peer affiliation and NSSI. That is, gratitude played an important role in reducing adolescents’ risk of NSSI.

## Discussion

Although stressful life events has been shown to be associated with adolescent NSSI (Cipriano et al., 2020; Gao et al., 2020; Steinhoff et al., 2020), the reasons for this association have been unclear. To address this research gap, we tested whether deviant peer affiliation mediated the relationship between stressful life events and NSSI, and whether this mediating effect was moderated by gratitude. The social development model (Hawkins and Weis, 1985) and the organism-environment interaction model (Cummings et al., 2002) informed our hypotheses. As expected, we found that deviant peer affiliation mediated the association between stressful life events and NSSI, and the indirect effects were weakened by adolescent gratitude.

### The mediating role of deviant peer affiliation

Consistent with hypothesis 1 and the social development model (Hawkins and Weis, 1985), we found that deviant peer affiliation mediated the relationship between stressful life events and adolescent NSSI. We consider this result with regard to the two parts of the mediated pathway: the link between stressful life events and deviant peer affiliation, and the link between deviant peer affiliation and NSSI. Tests of the first half of the mediation process (i.e., stressful life events → deviant peer affiliation) showed that stressful life events were positively associated with deviant peer affiliation among adolescents. Our finding is consistent with prior studies showing that parental conflict (Su et al., 2017), cyberbullying victimization (Chen et al., 2020), and peer rejection (Jiang et al., 2015) have all been shown to be associated with deviant peer affiliation. One potential explanation is that stressful life events increase adolescents’ psychological strain, and adolescents may respond to the psychological strain in a deviant manner (such as alcohol use). Based on the “interpersonal similarity principle,” adolescents may affiliate with peers who engage in the same or similar deviant behaviors (Gao et al., 2019). Another potential explanation is that for any number of reasons, adolescents join a group whose members show deviant behaviors, and are socialized to engage in deviant behaviors when they experience stressful life events.

Tests of the second half of the mediation process (i.e., deviant peer affiliation → NSSI) found that deviant peer affiliation was positively associated with adolescent NSSI, consistent with prior research (Wei et al., 2021). One potential explanation is that deviant peer affiliation may lead to adolescents’ problem behaviors, such as drinking (Sun et al., 2021), so these adolescents may experience more rejection and isolation by typical peers (Jiang et al., 2015). This in turn could lead to adolescents’ negative emotions such as depression (Platt et al., 2013). According to the integrated theoretical model of NSSI (Nock, 2010), NSSI has the function of regulating emotions such as depression, and adolescents may use NSSI as a means to relieve these negative emotions. In addition, multiple studies have shown that deviant behaviors, such as alcohol use (Sellers et al., 2021), drug use (O’Connor et al., 2014), and internet addiction (Lam et al., 2009) were positively correlated with NSSI among adolescents.

### The moderating role of gratitude

Guided by the organism-environment interaction model (Cummings et al., 2002), we tested the hypothesis that the individual difference of gratitude could weaken the relationship between risk environment and adolescent NSSI. Our findings were consistent with Hypothesis 2: the environmental risk factors were not as strongly linked with NSSI for youth with high gratitude. Specifically, high levels of gratitude offset the link between stressful life events and deviant peer affiliation, as well as the link between deviant peer affiliation and NSSI. These findings are consistent with those of previous studies (Zhou et al., 2014; Lo et al., 2017; Ma et al., 2022) that showed that gratitude as an important protective factor buffered the association between stressful life events and adolescents’ risk behaviors. Our results also support the view that gratitude plays an active role in growth following adversity (Wood et al., 2007).

Why might gratitude act as a buffer of the effects of risk? It is possible that adolescents with high gratitude will adopt more active strategies (such as positive reinterpretation and active coping) when facing stressful life events (Wood et al., 2007; Yu et al., 2010), and control their behavior in line with social expectations (He et al., 2018), which in turn could reduce deviant peer affiliation and NSSI. In addition, a large number of studies have shown that gratitude is positively associated with positive emotions, and negatively associated with negative emotions (McCullough et al., 2002; Watkins et al., 2003; Wood et al., 2007; Yu et al., 2010; Sun et al., 2019). Therefore, a high level of gratitude may increase adolescents’ life satisfaction (McCullough et al., 2002; You et al., 2018), thereby reducing the risk of deviant peer affiliation (Dou et al., 2020) and NSSI (Kress et al., 2015).

### Practical implications

The present study may have implications for preventing and treating adolescent NSSI. First, the results underscore the mediating effect of deviant peer affiliation on the association between stressful life events and adolescent NSSI. This pattern suggests that reducing the possibility of exposure to deviant peer group might be helpful to reduce the risk of adolescents engaging in NSSI behaviors. Therefore, it is necessary to take into account the risk factors of peers when formulating prevention and intervention strategies to reducing adolescents’ NSSI behaviors. Second, the results underscore that higher gratitude was a protective factor, which offset the relationship between risk environment (stressful life events and deviant peer affiliation) and adolescent NSSI. Therefore, interveners can improve the level of gratitude through intervention measures, including gratitude contemplation, gratitude lists, and expression of gratitude (for a review, see Wood et al., 2010), so as to protect adolescents from engaging in NSSI behaviors.

### Limitations and future directions

First, the data in the current study came from adolescents’ self-reports, which provide subjective information. Future studies can use reports from parents, teachers and peers to collect data. Second, this research was performed using a cross sectional study design, so inferred causality (what comes first, what follows) cannot be tested. Longitudinal designs should be considered in the future. Third, this study tested deviant peer affiliation as the mechanism of association between stressful life events and adolescent NSSI. Other mechanisms may also be salient, including self-compassion (Jiang et al., 2020) and school engagement (Yu et al., 2020). Fourth, in this study we tested the moderating effect of subjective reports of gratitude on the relationship between stressful life events and adolescent NSSI, but did not examine the impact of other subjective variables. Future research needs to examine the impact of multiple subjective variables, such as subjective reports of social support (Hu, 2022) and self-control (Zhu et al., 2021) on adolescent NSSI. Fifth, the influence of gender on adolescent NSSI was not explored in this study. There are gender differences in adolescent NSSI, with girls being more at risk than boys (Swannell et al., 2014; Rahman et al., 2021). Transgender adolescents are at especially high risk of NSSI (Jackman et al., 2016). Previous studies have shown that transgender adolescents are more subjected to peer victimization (Norris and Orchowski, 2020), and peer victimization is a risk factor for NSSI among adolescents (for a review, see Van Geel et al., 2015). Therefore, prevention and intervention for adolescent NSSI may need to take gender differences into account.

## Data availability statement

The raw data supporting the conclusions of this article will be made available by the corresponding authors, without undue reservation, to any qualified researcher.

## Ethics statement

This research was approved by the Ethics in Human Research Committee of the Department of Psychology, Guangzhou University. Written informed consent to participate in this study was provided by the participants’ legal guardian/next of kin.

## Author contributions

CW, YW, and CY conceived and designed the research. CW collected and analyzed the data. CW, TM, QZ, QX, HL, and ZL reviewed and edited the manuscript. All authors contributed to the article and approved the submitted version.
